# Achondroplasia in Latin America: practical recommendations for the multidisciplinary care of pediatric patients

**DOI:** 10.1186/s12887-022-03505-w

**Published:** 2022-08-19

**Authors:** Juan Llerena, Chong Ae Kim, Virginia Fano, Pablo Rosselli, Paulo Ferrez Collett-Solberg, Paula Frassinetti Vasconcelos de Medeiros, Mariana del Pino, Débora Bertola, Charles Marques Lourenço, Denise Pontes Cavalcanti, Têmis Maria Félix, Antonio Rosa-Bellas, Norma Teresa Rossi, Fanny Cortes, Flávia Abreu, Nicolette Cavalcanti, Maria Cecilia Hervias Ruz, Wagner Baratela

**Affiliations:** 1 Instituto Fernandes Figueira/Fiocruz, Av. Rui Barbosa, 716, 22250-020 Rio de Janeiro, Rio de Janeiro Brasil; 2 Unidade de Genética do Instituto da Criança, Hospital das Clínicas, Faculdade de Medicina, Universidade de São Paulo, São Paulo, São Paulo Brasil; 3 Hospital de Pediatria JP Garrahan, Ciudad Autónoma de Buenos Aires, Argentina; 4 Fundación Cardioinfantil – LaCardio, Bogota, Distrito Capital Colombia; 5 Universidade do Estado do Rio de Janeiro, Rio de Janeiro, São Paulo Brasil; 6 Unidade Acadêmica de Medicina, Hospital Universitário Alcides Carneiro, Universidade Federal de Campina Grande, Campina Grande, Paraíba Brasil; 7 Centro de Pesquisa sobre o Genoma Humano e Células-tronco, Instituto de Biociências, Universidade de São Paulo, São Paulo, São Paulo Brasil; 8 Faculdade de Medicina de São José do Rio Preto (FAMERP), São José do Rio Preto, São Paulo Brasil; 9 Grupo de Displasias Esqueléticas, Área de Genética Médica, Departamento de Medicina Translacional, Faculdade de Ciências Médicas da Universidade Estadual de Campinas, Campinas, São Paulo Brasil; 10 Serviço de Genética Médica, Hospital de Clínicas de Porto Alegre, Porto Alegre, Rio Grande do Sul Brasil; 11 Fundación para el Progreso de la Medicina, Córdoba, Córdoba Argentina; 12 Center for Rare Diseases, Clinica Las Condes, Santiago, Santiago Chile; 13 Servicio de Kinesiología Clinica Las Condes y Universidad de Los Andes, Santiago, Santiago Chile; 14 Faculdade de Ciências Médicas da Santa Casa de Misericórdia de São Paulo, São Paulo, São Paulo Brasil

**Keywords:** Dwarfism, Management, Medical practice, FGFR3, Bone dysplasia, Guideline

## Abstract

**Background:**

Achondroplasia is the most common bone dysplasia associated with disproportionate short stature, and other comorbidities, such as foramen magnum stenosis, thoracolumbar kyphosis, lumbar hyperlordosis, genu varum and spinal compression. Additionally, patients affected with this condition have higher frequency of sleep disorders, ear infections, hearing loss and slowed development milestones. Considering these clinical features, we aimed to summarize the regional experts’ recommendations for the multidisciplinary management of patients with achondroplasia in Latin America, a vast geographic territory with multicultural characteristics and with socio-economical differences of developing countries.

**Methods:**

Latin American experts (from Argentina, Brazil, Chile and Colombia) particiáted of an Advisory Board meeting (October 2019), and had a structured discussion how patients with achondroplasia are followed in their healthcare centers and punctuated gaps and opportunities for regional improvement in the management of achondroplasia.

**Results:**

Practical recommendations have been established for genetic counselling, prenatal diagnosis and planning of delivery in patients with achondroplasia. An outline of strategies was added as follow-up guidelines to specialists according to patient developmental phases, amongst them neurologic, orthopedic, otorhinolaryngologic, nutritional and anthropometric aspects, and related to development milestones. Additionally, the role of physical therapy, physical activity, phonoaudiology and other care related to the quality of life of patients and their families were discussed. Preoperative recommendations to patients with achondroplasia were also included.

**Conclusions:**

This study summarized the main expert recommendations for the health care professionals management of achondroplasia in Latin America, reinforcing that achondroplasia-associated comorbidities are not limited to orthopedic concerns.

## Background

Among the skeletal dysplasias associated with short stature, achondroplasia is the most common disorder, affecting nearly 1 in every 27,000 live births with prevalence ranging from 0.36 to 0.6 (CI 0.30 – 0.74) per 10,000 births in the USA while in South America the prevalence is 0.44 (CI 0.33 – 0.55) per 10,000 births [1, 2]. Although the majority of studies investigating the prevalence of achondroplasia are population- or hospital-based assessments [3], these numbers translate that achondroplasia affects at least 250,000 persons worldwide [4]. It is caused by abnormal endochondral ossification of the skeleton due to common gain of function mutation in the fibroblast growth factor receptor 3 (FGFR3) gene [5]. There are mainly two variants (c.1138G > A and c.1138G > C) leading to the same amino acid change in position 380 of the FGFR3 (p.Gly380Arg) found in more than 95% of the affected individuals. FGFR3 exerts a negative effect in the growth plate chondrocyte proliferation and terminal differentiation; these variants cause an exacerbation of this physiological function, impairing linear bone growth [5].

Achondroplasia is characterized by disproportionate short stature, macrocephaly, facies with frontal bossing and midface retrusion, exaggerated lumbar lordosis, genu varum, brachydactyly, trident appearance of the hands and joint hypermobility [6, 7]. The average adult height for patients with achondroplasia is 128 cm for men and 120 cm for women in regional growth data [8] similar to other references [9–11].

Individuals with achondroplasia may have craniocervical junction compression on imaging and a small proportion of them may display neurologic and respiratory complaints [3]. Studies have consistently demonstrated both higher frequency of sudden death and higher mortality in children younger than 4 years old due to neurological complications, respiratory failure and accidents [12, 13]. In addition, these patients present with midface hypoplasia, adenotonsillar hypertrophy and stenosis of upper airways, contributing to sleep-breathing disorders. Other associated features are obesity, delayed motor milestones, speech delay, high frequency of otitis media and bowing of the lower legs [14].

Patients with achondroplasia are at risk of several complications and impairments in their functional capacity and quality of life [15]. Many medical complications associated with achondroplasia are related to the disproportion between endochondral growth of the bones and organ tissues. The modified recombinant human C-type natriuretic peptide analogue vosoritide (Voxzogo™, BioMarin Pharmaceutical Inc, Novato, CA) has been recently approved in several countries as a pharmacological therapy for children with achondroplasia. Brazil was the first country in South America to obtain approval for use by the regulatory agency.

In Latin America, there are significant unmet needs in the follow-up of patients with achondroplasia. Barbosa-Buck et al. [2] systematically evaluated the prevalence of skeletal dysplasias in more than 1.5 million births in South America from 2000 to 2007. These authors verified an elevated paternal age (34.9 ± 7.8 *p* = 0.001) and a mutation rate per gamete per generation of 1.74 (1.25 – 2.25) × 10^–5^. An early and efficient diagnosis and evaluation of the patients are warranted as well as adequate genetic counselling.

Latin America is a vast geographic territory with multicultural characteristics and with socio-economic diversity. The health care systems are different in each country and also inside them. Besides the geography, there is also a lack of specialists in rare diseases. Within this context, the present study is dedicated to summarizing recommendations for the multidisciplinary management of achondroplasia patients suggested by experts in achondroplasia from Latin America that might assist clinical management of pediatric individuals with achondroplasia.

## Methods

Although its clinical and radiologic features have been well known for more than 50 years, there are still controversies about the ideal clinical management to investigate and treat achondroplasia-related medical issues, such as cervicomedullary compression, sleep apnea, spinal stenosis and mental and psychological health issues. Therefore, the present work was drawn upon the clinical experience of a Latin American healthcare expert panel and published peer reviewed articles to sustain practical recommendations for the general and multidisciplinary care of pediatric patients with achondroplasia in the context of Latin America.

Latin America experts (from Argentina, Brazil, Chile and Colombia) were brought together in a face-to-face meeting to discuss gaps and opportunities for regional improvement in the management of achondroplasia. The Advisory Board was held in October 2019, in São Paulo and was sponsored by BioMarin Farmacêutica do Brasil LTDA. The key takeaways are summarized in the present work.

## Results

### Practical recommendations for the multidisciplinary care of pediatric patients with achondroplasia

#### Role of geneticist (genetic counselling)

In nearly 80% of patients, achondroplasia arises as a de novo disease-causing variant Gly380Arg in *FGFR3* gene. Because of that, the same reproductive partners have a very low risk of having another child with the same condition. In these circumstances, it is well demonstrated that there is a positive correlation between the risk for dominant de novo mutations associated with advanced paternal age [16, 17]. All mutations are paternal in origin [18].

**Fig. 1:**
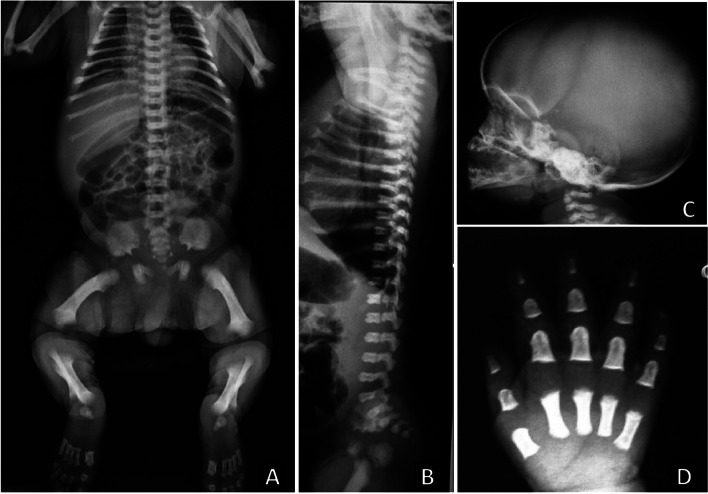
Radiographic images of achondroplasia in the newborn period. **A** Generalized platyspondyly leading to an increased height of the intervertebral spaces. The pelvis shows a squared configuration with a horizontalized acetabular roof and narrowness of the sciatic notch. Pubis and mainly ischia are broad. The long tubular bones are shorter with metaphyseal irregularity and the fibulae are greater than tibia. Note the oval-shaped lucent appearance of the proximal femora. **B** In the lateral view, it is also evident an increased lumbar lordosis. **C** The cranium is large and both frontal and occipital regions are prominent. **D** The hand show shortening of all tubular bones – metacarpal and phalanges

Molecular testing of *FGFR3* variants can be performed to confirm the radiological diagnosis [9] and there is the possibility of testing for a multi gene NGS (next-generation sequencing) panel in the case of differential diagnosis among skeletal dysplasias [7]. More importantly, radiographic baby gram evaluation per se can provide valued information in all cases suspected of having a bone dysplasia [7, 19], Fig. 1.

Genetic counselling is indicated in all cases and should include discussion of the diagnosis, natural history of the disease and recurrence risk. In the case neither parent has achondroplasia, the recurrence risk is low, however if one parent is affected there is a 50% recurrence risk. If both parents have achondroplasia, the offspring’s risk of having the same condition is 50%, 25% of having average stature and 25% of having homozygous achondroplasia. In the later scenario, homozygous or compound heterozygous for disease-causing mutations lead to a far more severe condition, almost always life-limiting [19, 20]. In addition, it is not uncommon for people with achondroplasia to have partners with other diagnoses of skeletal dysplasia. Thus, genetic counselling may be useful to discuss the overall risks of recurrence, not only for two parents with achondroplasia, but also for two parents with different skeletal dysplasias.

Another important role of the genetic counselling is to clarify the need for molecular analysis and necropsy in cases of miscarriage, stillbirth, or termination of pregnancy. Furthermore, genetic counselling can provide orientation for the family decision of pregnancy continuation. Ideally, any woman affected with achondroplasia, or any other skeletal dysplasia, should be genetically counseled as part of the genetic counselling program. Additionally, the international consensus statement on the diagnosis, multidisciplinary management, and life-long care for individuals with achondroplasia suggests that the counselling should include a discussion of postnatal management and prognosis [21].

#### Role of gynecologists and obstetricians in prenatal diagnosis and planning of delivery

Achondroplasia is generally suspected around 24–25 weeks of gestation using routine ultrasound [22–24] although prenatal diagnosis of skeletal dysplasias may be challenging due to phenotypic variability [25]; indeed, the accuracy of prenatal ultrasound in detecting a specific skeletal dysplasia has been reported as 40 to 60% [26]. Ceroni and colleagues [6] reported the natural history of 39 patients with achondroplasia followed in a reference center in Brazil. These authors showed that 67% of patients were diagnosed prenatally or at birth, while 33% received diagnosis within the first year of life. These data pointed out the importance of disease awareness by gynecologist, obstetrician and pediatricians. Below are some characteristics and recommendations to facilitate diagnosis:

- Fetuses with achondroplasia display shortening of the long bones (femora or humeri length measurements less than 5th centile or -2 standard deviation (SD) from the mean in the second trimester) [27]. Macrocephaly, frontal bossing and widened femoral diaphyseal-metaphyseal angle [23] may also be visualized during ultrasound evaluation.
- Achondroplasia should be suspected when shortened long bones are noted on the third trimester ultrasonography [28].
- Complementary evaluations such as fetal Magnetic Resonance Imaging (MRI) may constitute an interesting complementary tool, assisting in the evaluation of the central nervous system, spine and pulmonary volume. In addition, MRI may also assist in decision making for amniocentesis, choice of genetic testing and delivery planning [22].
- Any signs that could contribute to the detection of lethality should be evaluated, such as thoracic cage versus abdominal circumferences ratios in the prediction of pulmonary hypoplasia, visceral abnormalities, polyhydramnios and hydrops fetalis [27, 29].
- The anesthesia procedure and the delivery are critical to ensure safety to both mother and her baby [30]. For non-achondroplasia pregnant women, psychological follow-up and genetic counselling are critical as well as the evaluation of the fetus regarding macrocephaly, hampering the vaginal delivery. Regardless of the offspring being affected or not, pregnant women with achondroplasia should deliver by cesarean section because of a small pelvis-to-head size ratio in the fetus [31], Fig. 3-A. However, based on clinical circumstances, the international consensus has recently proposed that vaginal delivery may be suitable when under the care of an experienced obstetrician [21].

### Practical recommendations to pediatric follow-up of achondroplasia according to development phases

#### Perinatal period

- During delivery, there is a need for neonatologist because some babies display respiratory compromise in the immediate newborn period.
- Diagnosis should be confirmed by both clinical and radiologic evaluation after delivery. Proximal shortening of limbs in at least the arms, large head, narrow chest and short fingers with “trident” shape are signals that should confirm the prenatal suspicion [3, 19]. According to Spranger et al., [32], radiographic features are: large calvarium, decrease in the interpedicular distance from upper to lower lumbar spine, short pedicles in the lateral view of spine. In infancy (aged 0 to 5 years), square or oval radiolucent areas in proximal femur are also observed.

#### Growth development

- Height and weight should be measured at each pediatric visit and followed according to specific growth chart [33, 34].
- Carefully measure head circumference during the first two years of age with specific growth charts [8, 33]. If growth is excessively high, refer for neurosurgery evaluation.
- Development evaluation: in infants, hypotonia and delayed acquisition of motor patterns are common. Usually, there is spontaneous improvement with age. Most children develop motors skills later, sitting without support about the age of 14 months and walk independently around 19 months [35]. Additionally, expressive language delay, most due to unrecognized persistent or fluctuating hearing loss, normalizes by 5 or 6 years of age in the vast majority of children with appropriate speech and language therapy [3, 36].
- Young children with achondroplasia demonstrate a number of unique movement strategies apparently compensatory for the biomechanical changes. It is suggested to use control condition-specific developmental profile as published by Ireland et al. [20, 35].
- Respiratory evaluation: upper airway is often narrow and children at all ages have snoring. The snoring is frequent and subsides with the change of position allowing a peaceful sleep. When the tightness is greater, obstruction can occur that alters sleep and diet. Sleep studies are highly recommended for children with achondroplasia due to obstructive sleep apnea and central sleep apnea. In obstructive sleep apnea, some signs are observed as increased retraction, glottal stops, choking, intermittent breathing, apnea, deep compensatory sighs, secondary enuresis, recurrent nighttime awakening or emesis. The central apnea results from compression of the craniocervical junction and arteries at the level of the foramen magnum. Prolonged apnea while sleeping is associated with the risk of craniocervical junction. Significant compression can also result in worsening of obstructive apnea as it can cause a decrement/reduction in the infant’s airway tone [37].
- Counsel parents on how to help preventing kyphosis. Within this context, Pauli et al. [38] strongly advise avoidance of unsupported sitting in order to prevent gibbus. In addition, certain types of children carriers, mechanical swings, jumpers and umbrella-style strollers should also be avoided.

##### Recommendations related to the neurologic concerns

Major concerns in a patient with achondroplasia are craniocervical junction constriction and restrictive pulmonary disease [3, 7], which potentially exposes the child to sudden death. Indeed, the mortality rate in the first year of life in children with achondroplasia is 41.4 in each 1.000 live births [39, 40].

Narrowing of foramen magnum in infants and toddlers can create acute or chronic injury to the spinal cord. The chronic injury is more frequent and occurs in about 10 to 20% in infants and toddlers. Neurological symptoms are clonus, hyperreflexia, asymmetric tone, weakness, poor suck, failure to achieve development milestones and sleep disorders [41].

- In accordance with the American Academy of Pediatrics’ guideline on health supervision for children with achondroplasia [19], Computed Tomography (CT) or MRI of the cervical-cranium level are recommended to screen all patients with achondroplasia. In 2015, an experience-based recommendation from eleven multidisciplinary international experts on skeletal dysplasia verified that screening all patients for foramen magnum stenosis did not reach the minimum consensus rate (80%) among the specialists [41]. The panel concluded that universal screening of infants with achondroplasia by these neuroimaging methods is still a matter of debate. On the other hand, a recent international consensus suggested MRI scanning to evaluate the cervicomedullary junction and foramen magnum size should be considered during the first months of life, either in asymptomatic patients with achondroplasia [21]. In Latin America, access to CT or MRI of the cervical-cranial region is limited by economic and geographic reasons, and there are limitations in the possibility of offering these evaluations even in reference centers. Considering these limitations, White et al. [41] also recommended that, due to the risk of managing achondroplastic patients during anesthesia, a decision for MRI should only be taken after sharing the clinical findings with a multidisciplinary team and in accordance with the neurologist/neurosurgeon. Clinical signs during the neurologic examination of young achondroplastic patients as clonus, hyperreflexia, asymmetric tone, weakness, poor suck, failure to achieve developmental milestones and sleep disorders can guide the need of CT/MRI studies [3, 42].
- Foramen magnum decompression is indicated in symptomatic children with cervicomedullary compression, with or without signal changes in the spinal cord, and ideally should be carried out by a neurosurgery team with experience in this procedure in achondroplastic patients.
- Hydrocephalus in patients with achondroplasia remains controversial. In patients with achondroplasia, the association between macrocephaly and the ventriculomegaly (that leads to an excessive extra-axial fluid) might be misinterpreted as hydrocephalus [43, 44]. Patients rarely have signals and symptoms related to hydrocephalus. In these cases, the placement of a ventriculo-peritoneal shunt is recommended [3]. In addition, screening with ophthalmology is recommended in order to search for optic nerve edema (papilledema) as an indirect signal of hydrocephalus.

##### Recommendations related to the orthopedic concerns

**Fig. 2:**
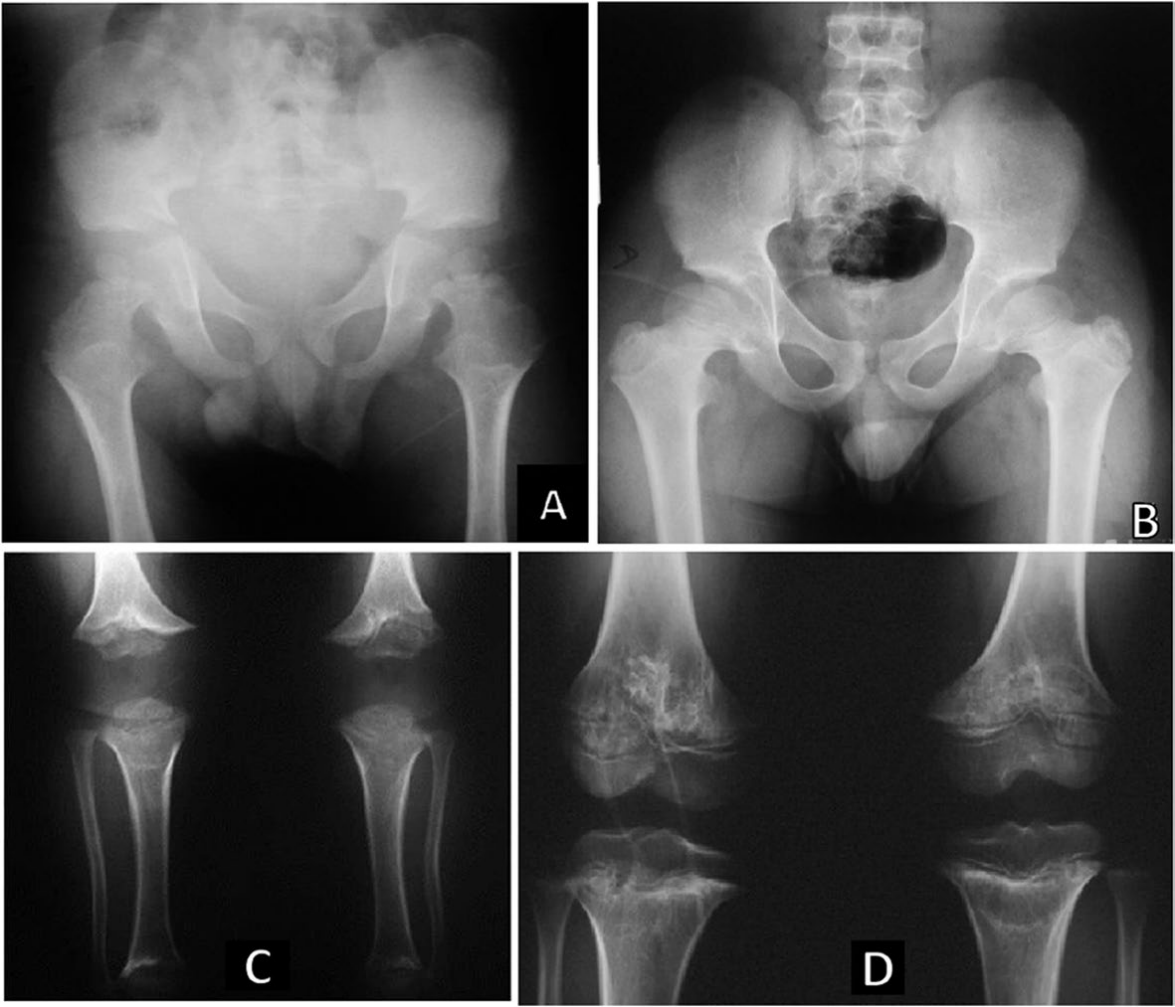
Radiographic images of children with achondroplasia at the ages of 4 (**A** and **C**) and 8 years (**B** and **D**). **A**-**B** Note squared configuration of the pelvis that remains with the years, as well as the narrowness of the sciatic notch. The femoral neck became short. In the lower long bones, the irregularity of metaphysis of the knees shows, with the age, the “chevron” shape of the distal region of femora. **C**-**D** The tibia is broad and the fibulae greater than the tibia

**Fig. 3:**
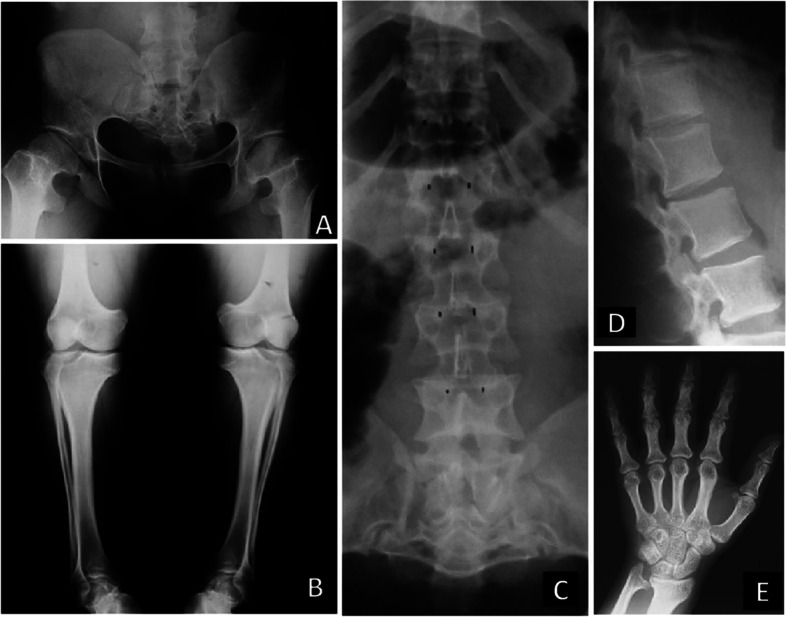
Radiographic images of adults with achondroplasia. **A** The pelvis shows the same squared shape observed in the early ages, and now the femoral neck is more evident. **B** In the knees, we can observe the epiphyseal region as broad as metaphysis. Tibiae and fibulae are shorter, however, this last one is longer than the tibiae. **C** The narrowing of the interpedicular space in the lumbar region is evident in the anteroposterior incidence of the spine X-ray. **D** In the lateral view of the spine, it can see the concave aspect of the posterior margin of the vertebral bodies. **E** The hands are short and broad and the ulnar styloid process is prominent

- The American Academy of Pediatrics [19] has recommended postnatal radiographic follow-up evaluations with parsimony. The American experts defend the idea that children with achondroplasia are excessively exposed to X-ray radiation. To the best of their experience, radiographic evaluation is a great tool in two situations: a) when achondroplasia was not detected during gestation and there is clinical suspicion of the condition [37] and b) when there is the necessity of surgical correction of any orthopedic issue, like kyphosis. Figures 1, 2 and 3 show the main bone changes observed in patients with achondroplasia at different stages of development.
- Transient kyphosis in the thoracolumbar junction is present in most infants with achondroplasia under one year of age [38, 42, 45]. In general, observation of kyphosis accompanies sitting transition [46]. Pauli [3] has proposed that appearance of kyphosis is related to hypotonia, macrocephaly and ligamentous laxity, causing a C-sitting posture. Within this context, Trotter and Hall [19] strongly advise avoidance of unsupported sitting in order to prevent gibbus. In addition, certain types of children carriers, mechanical swings, jumpers and umbrella-style strollers should also be avoided. There is a small group of patients who will develop a fixed kyphosis.
- When the patient is older, physical activity should be strongly encouraged. In older children (2–3 years of age), hyperlordosis is nearly universal and usually asymptomatic, with no treatment required.
- Genu varum is a prevalent orthopedic concern not only in children but also in adults with achondroplasia [47]. Its etiology may be related to lateral instability, coxa vara and fibular overgrowth. Pain in knees is not related to deformity, but to hypermobility. Possible osteotomy for correction may be performed when the child is nearly at eight years of age.
- Although genu varum may be present in many patients (approximately 60–7-%), some may have normal alignment or increased genu valgus. Since hypotonia and increased laxity is a frequent finding in these patients, instability of the knee can be present (lateral thrust). If genu varum is present after the age of 2 years, weight bearing X-rays of the lower extremities should be taken. If there is pain or instability, surgical treatment should be considered; osteotomy of the tibias may be needed on some occasions. Guided growth technique is also an option, and the procedure should be performed before age of  5 years (osteotomies and limb lengthening can be performed simultaneously) [48, 49].
- Indications for limb lengthening in achondroplasia are controversial. Lower limb lengthening in a selected group of patients with achondroplasia can increase height, improve body proportion, functionality, self-esteem and quality of life. Lengthening of the humeri can improve independence and personal hygiene [3, 50–53].
- Bilateral humeral lengthening improves independence and body proportion in patients with achondroplasia [54–57].
- Flexion deformity of the elbow is a frequent finding in patients with achondroplasia and can be corrected simultaneously while lengthening [58].

##### Recommendations related to development milestones

Psychomotor delays in infants with achondroplasia have been frequently observed [42, 59–61]. Such delays in development have been attributed to a relatively large head, ligamentous laxity and body disproportions. Even though delayed development milestones for gross motor and communication were reported, fine motor skills are less evaluated. Nevertheless, the international consensus pointed to differences in fine and gross motor development milestones in achondroplastic children when compared to average stature, age-matched peers, and suggested follow-up using specific screening tools. If pattern changes are observed, MRI of head and spine and an evaluation by a pediatrician, and/or neurologist should be considered [21].

- We propose a trimestral evaluation of development milestones by the pediatrician during the first year of life and the subsequent evaluations could be performed annually using condition-specific developmental charts [20, 35].

##### Recommendations for nutritional aspects and anthropometry

Obesity has been recognized as a medical health problem in achondroplasia, possibly aggravating orthopedic and neurologic problems (lumbar spinal stenosis, joint pain), as well as obstructive sleep apnea [62]. However, there is a lack of data regarding the ideal oral intake in infants with achondroplasia and other dwarfism diseases or clear information about the appropriate weight gain velocity in these patients [63].

It has been demonstrated that the most frequent disease-causing variant (Gly380Arg – for review, see Xue et al., [64]) lead to decreased proliferation of chondrocytes [65] and simultaneously to an enhanced adipocyte differentiation, resulting in a strong predisposition to abdominal obesity [62].

Interestingly, achondroplasia-induced orthopedic features may affect both caloric intake and energy expenditure, increasing the risk for obesity. As the ribs of patients with achondroplasia are shorter, the thoracic cage is smaller and, consequently, lungs are also smaller. In addition, because of laxity, ribs are more flexible, resulting in a paradoxical movement with inspiration and decreased effective inspiratory volume. Patients experience tachypnea and have additional energy expenditure while breathing [66]. In association with tachypnea, hypotonia may also contribute to a decreased capacity to drink or swallow properly, leading to gastroesophageal reflux disease [7, 19].

Anthropometric curves for patients with achondroplasia from different ethnicities are available in the literature but seem to be infrequently utilized outside specialty clinics [9, 10, 67].

- Children with achondroplasia experience a period of fast decreasing growth during infancy with a similar shape but lesser in magnitude compared to general population.
- Shifts in growth channels, between birth to 5 year of age, may be seen in 48.8% of children with achondroplasia and professionals who follow up them must consider this phenomenon during infancy and early childhood.
- Use of adequate anthropometric curves is critical in order to properly evaluated both growth and weight gain in each consultation [8, 33, 34].

The anthropometric evaluation of adults can also be performed using the current available curves [3]. It should be noted that the use of body mass index to average individuals can inappropriately define patients with achondroplasia as obese because of body proportion differences [3]. Dietitians should propose a dietary plan that preconizes lower weight gain velocities in order to prevent both overfeeding and underfeeding [63].

##### Recommendations for otorhinolaryngologic evaluation

The frequency of sleep disorders varies from 10 to 87% in children with achondroplasia [68]. This concern is so critical that the American Academy of Pediatrics recommends performing neuroimaging and polysomnography (PSG) at the time of diagnosis and in cases of suggestive symptoms of sleep disordered breathing [37].

Achondroplasia anatomic facial alterations, such as midface hypoplasia, micrognathia, depressed nasal bridge, choanal stenosis, high palate and decreased temporo-mandibular joint mobility may critically contribute to development of upper airway obstruction [69]. Moreover, these children may also present macroglossia, tracheobronchomalacia and physiologic hypertrophy of adenoids and tonsils at age of 2 years approximately (reflected by adenoids and tonsils hypertrophy) [70]. All of these collectively with a physiologic decreases muscular tone during sleep, which leads to a narrower airway [71], are the most likely contributors to pathogenesis of obstructive sleep apnea in children with achondroplasia.

It has been reported that airway obstruction can also be centrally mediated. In this way, Bagley and colleagues [72] investigated the effects of surgical decompression in children with achondroplasia. These authors verified that the surgical procedure reduced sleep apnea in that cohort. In agreement, Baujat et al. [73] proposed that cervical cord compression could be a major cause of central sleep apnea. The involvement of foramen magnum stenosis had been previously studied by Tasker and coworkers [74]. This group proposed that the upper airway obstruction may not be induced by foramen magnum stenosis in all cases. In fact, the cranial nerves that control muscles of pharynx, larynx, and upper part of the oesophagus leave the cranium via the jugular foramen not via foramen magnum, suggesting that the involvement of the foramen magnum stenosis is related to the degree of cranium dysplasia.

- Sleep studies should be performed in the first year of life, or at the first sign of breathing disorders.
- In centers where sleep studies are limited, the evaluation with oximetry may assist in selecting the most urgent cases.
- The impact of sleep apnea on cognitive performance should be considered [3]. In this regard, Hecht and colleagues [61] have found a correlation between severe respiratory dysfunction with abnormal polysomnography and a lower intellectual potential in 13 achondroplastic infants.
- Patients with achondroplasia usually require otolaryngologic surgery such as adenoidectomy and/or tonsillectomy, as well as ventilation tube placement surgery [75].

Another otorhinolaryngologic concern is the middle ear dysfunction, which is observed both in children and adolescents with achondroplasia. The proposed mechanism is of the poor functioning and abnormally oriented Eustachian tubes, which may be derived from aberrant growth of the chondrocranium [3]. Although there is a lack of a well-structured prospective study, there is evidence that the frequency of otitis media is nearly 50–70% of individuals with achondroplasia [76, 77].

- Patients with achondroplasia may present with at least one episode of otitis media during the first year of life and almost 90% had an episode by the age of two years [78]. Thus, hearing assessment and tympanometry should be performed before the age of one year and should be annually repeated. Moreover, hearing evaluation should be performed in case of suspicion of hearing loss [19, 37].
- Nearly half of the patients with achondroplasia are subjected to the placement of pressure equalizing tubes throughout their infancy [77]. Pediatricians need to be aware of the occurrence of jugular bulb dehiscence into the middle ear space due to abnormalities of the chondrocranium [79].
- Hearing impairment may be suspected during the language acquisition period, constituting a factor in speech and language delay [3, 19, 37].

##### The role of physical therapy and physical activity

Physiotherapy involvement should be part of the health supervision plan and would include prevention of secondary complications and targeted education. Physiotherapist should educated caretakers about appropriate physical activities, the importance of avoiding unsupported sitting (in order to avoid gibbus) and different positions to use during rest and periods of play. Parents should be informed early on how to position the children, specially to avoid sitting without head support until adequate head control is achieved.

If the child is left without any intervention, there is a risk of developing a marked spine flexion, with a possible wedging of vertebrae, causing fixed thoracolumbar kyphosis.

In adults, lumbosacral spinal stenosis is a very common problem. They should be instructed to perform periodic clinical examinations and avoid jobs/functions that require prolonged maintenance of standing posture or that perform trunk flexion /extension.

- The choice of physical activity must be careful in children and adolescents with achondroplasia. Sports like swimming and cycling are encouraged to improve strength, coordination and expend calories. Conversely, sports with emphasis in force, physical contact or jumps should be avoided to reduce undesirable stress on the joints.

##### The role of phonoaudiology

- Delayed language acquisition is common and consequently there should be an annual evaluation with referral to language therapy if needed.
- As cognitive delay may be related to delayed language acquisition, it is recommended that all children with achondroplasia should be screened by tools proposed by Ireland and colleagues [35].

#### Scholar years

During school years, the inclusion of children in school may require environmental modifications and psychological follow-up. Children should be taught about their condition to improve acceptance and self-esteem and should be encouraged to have as similar lifestyle as possible as an average-stature child.

##### Recommendations to increase physical function and quality of life

- Environments at home and school should be adapted to ensure the independence of children with achondroplasia, such as toilets, chairs (so that children can sit without dangling legs and an unsupported back), clothes (hemmed), etc. [3, 19].
- Occupational therapists can provide support in adapting different environments and helping children manage on a daily basis [80].
- Parents should be advised of support groups for patients and families.
- Personal perineal hygiene can be a daily issue for patients with achondroplasia. As detailed by Pauli [3], children can be taught to hop off the toilet, and bend far forward, reach between the legs and wipe front to back [3].

##### Physical activity

- Physical activity such as swimming, biking, water aerobics, tennis and soccer are safer activities and should be encouraged to improve strength and coordination [3]. On the other hand, parents should be counseled about the risks of activities that may lead to trauma-based cord compression, such as gymnastics and collision sports. In soccer, heading should be prohibited [19].

##### Quality of life of the patient and families

There is a lack of studies investigating the quality of life in children with achondroplasia. Studies with adults affected by this condition, revealed that 2/3 of patients reported chronic pain, which is associated with reduced physical function and limited independence [81]. It has been reported that individuals with achondroplasia have diminished income, less education and less successful employment, resulting in lower resilience [82].

- Quality of life can be assessed in older children and young adults by the APLES (Achondroplasia Personal Life Experience Scale) [83, 84].

It has been demonstrated that children with chronic illness and their families have decreased quality of life [85]. Witt et al. [86] demonstrated that parents of children with achondroplasia feel overwhelmed when they receive the diagnosis, regardless of the time (pre or postnatal).

- The impact on the quality of life of parents and caregivers can be reduced when they participate in a patient organization as employees or volunteers, since sharing experience can help developing coping strategies [86].
- Parents and/or caregiver should be routinely assessed for physical and mental stress and interventions can be done to promote parents and/or caregivers’ adaptations [87].
- Parents can be referred to psychologists and psychiatrists in order to discuss physical stress, stigmatization, feelings of guilt and fears of the future that they can feel [88, 89].

##### Growth

Studies of growth with a longitudinal design show that children with achondroplasia experienced a period of fast growth during infancy followed by a smooth and continuous decrease to nearly linear growth in the preschool period [34]. During adolescence, they show a period of rapid increase of height with a later slowdown known as the “adolescent growth spurt” [90]. The height is approximately 5.00 SD below the median compared non-achondroplastic populations with a mean adult height of approximately -6 SD [8, 10, 11, 91].Thus, the curves are similar in shape but lesser in magnitude in comparison to the general population in both periods [34, 90]. Besides, 72% of that small growth peak is caused by the peak of trunk length [90, 92, 93].

With respects to segmental growth, lower limbs are affected early prenatally and become more noticeable throughout their lifetime. In opposition, growth of the trunk is not affected as severely as limbs. The disharmonic growth between near normal trunk and the severe growth retardation of limbs determine body disproportion in achondroplasia [33].

Physical pubertal changes, such as occurrence of breast bud or increase in testis size, are related with periods of fast growth observed in adolescence. In boys, genital development 1 and 5 and testicular volumes of 4 and 20 ml, which represent the beginning and the end of pubertal development, appears before and following the age at peak height velocity respectively. In girls, menarche occurrs, like non-affected girls, following the age at peak height velocity and towards the latter part of secondary sexual development, i.e. in breast stage 3, 4 or 5 [90].

#### Transition to puberty and to adult life

Throughout an achondroplastic patient’s life multiple phases of distinct medical needs and psychosocial adaptations are required, creating anxiety for the patient and family. Transition to puberty and then to adult life, with professional and mating issues, also bring distinct physical, medical and psychological needs from those of infancy.

##### Sexual life and the role of genetic counselling

- Management of birth in achondroplastic women should be individualized. Delivery beyond 32 weeks should be by cesarean section and the anesthetic procedure should be analyzed case by case to define the best option [94]. Preterm infant delivery before 32 weeks can be defined based on the cephalopelvic proportion [21], and the vaginal delivery may be indicated depending on clinical circumstances and considering the experience of the team.
- Before getting pregnant or planning pregnancy, all patients with achondroplasia should be subjected to genetic counselling in order to discuss the risk for the offspring to present the same phenotype (50%) and in cases in which the partner also has achondroplasia, the risk for biallelic variants in *FGFR3*, which is potentially lethal.

##### Recommendation in perioperative management

Achondroplastic patients have an increased risk for perioperative complications related to the anatomy of their upper airway, abnormal mobility of the upper cervical spine and body habitus [41]. Due to abnormal upper airway morphology, patients with achondroplasia may present difficult in intubation. The recommendations include a perioperative neurological, pulmonary and anesthetic evaluation.

A statement from international consensus established that these patients, especially children, should be anesthetized in hospitals preferentially where care providers are knowledgeable and experienced in caring for achondroplastic patients [21].

### Strengths and limitations

**Fig. 4:**
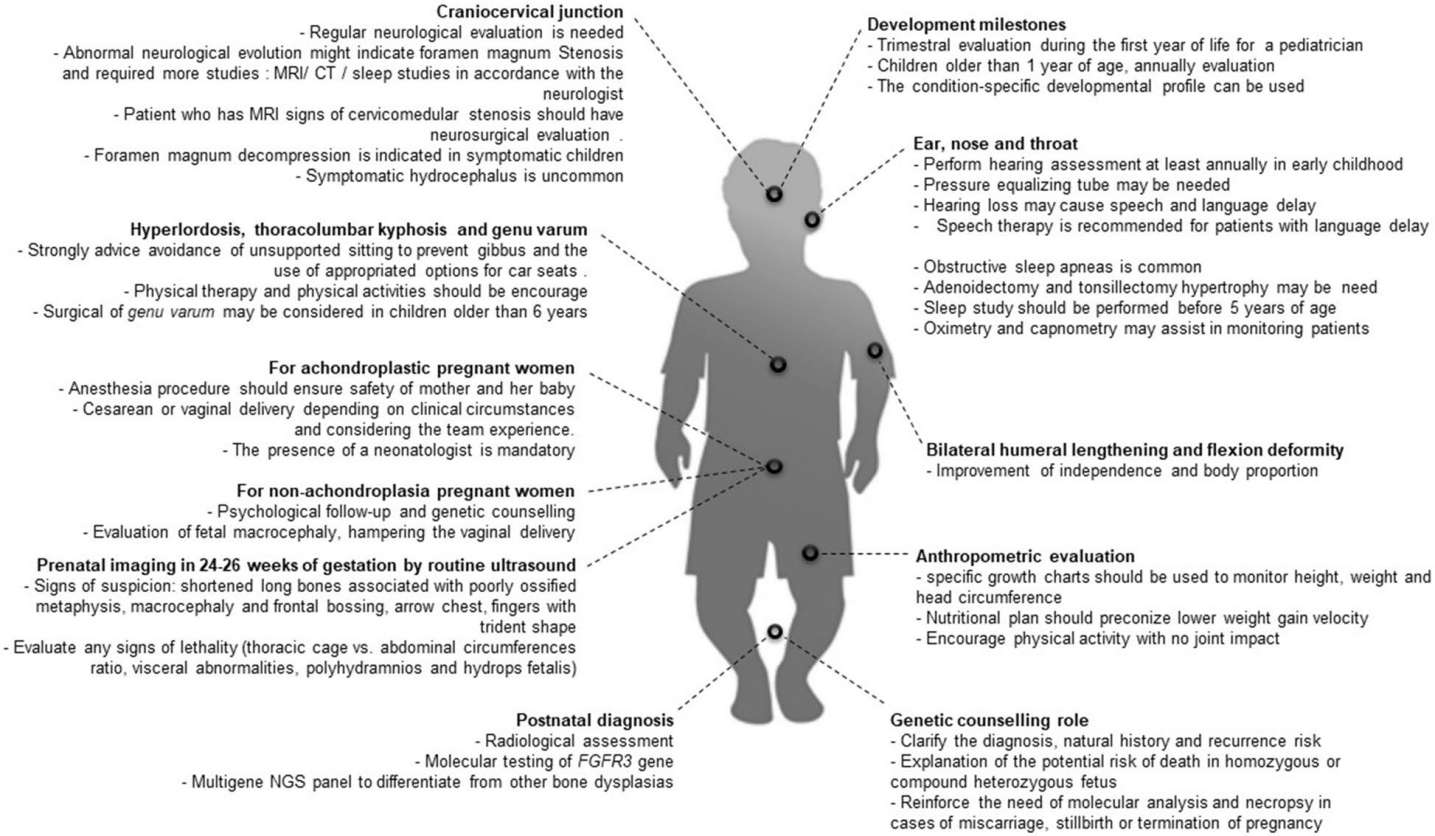
Summary of the proposed recommendations for the multidisciplinary management of achondroplastic patients. MRI: Magnetic Resonance Imaging, CT: Computed Tomography

Our work summarized the hallmarks of achondroplasia-associated comorbidities. In this scenario, it is clear that patients would potentially benefit from being treated for multidisciplinary teams and/or in reference centers. Although the proposed recommendations here are experts’ experience-based, there is a lack of evidence from each recommendation. The summary of the proposed recommendations is shown in Fig. 4.

## Conclusion

Even though achondroplasia is the most common non-lethal skeletal dysplasia and follow-up with the orthopedists is undoubtedly important, it is undeniable that disease-associated comorbidities are not limited to orthopedic concerns, thus requiring a multidisciplinary team of health care professionals to deal with distinct necessities. We presented an outline of strategies to treat patients with achondroplasia in Latin America, a vast geographic territory with multicultural characteristics and with socioeconomic limitations of developing countries.

## Data Availability

All data generated or analysed during this study are included in this published article.

## Abbreviations

CT: Computed tomography
FGFR3: Fibroblast growth factor receptor 3 gene
MRI: Magnetic resonance imaging
NGS: Next-generation sequencing
SD: Standard deviation
